# Lymph node yield as a measure of pancreatic cancer surgery quality

**DOI:** 10.1016/j.sipas.2022.100103

**Published:** 2022-07-09

**Authors:** Kevin Zhao, Ayobami Fatunmbi, Shengxuan Wang, Katelyn Young, Rebecca L. Hoffman, Joseph A. Blansfield

**Affiliations:** a Geisinger Commonwealth School of Medicine, 525 Pine St., Scranton, PA 18510, United States; b Geisinger Medical Center, 100 N. Academy Ave., Danville, PA 17822, United States

**Keywords:** Pancreatic adenocarcinoma, Lymph node harvest, Neoadjuvant therapy, Evaluated lymph node yield, Surgical quality improvement, Perioperative outcomes

## Abstract

**Introduction:**

Evaluated lymph node (ELN) yield has been established as a promising measure of surgical quality. Research has suggested that an ELN of at least 15 in pancreatic cancer patients is associated with improved survival and staging metrics. The aim of this study was to determine what impact a high ELN yield of ≥15 has in a novel population.

**Methods:**

A retrospective cohort study was performed of patients with resectable, non-metastatic pancreatic adenocarcinoma who underwent neoadjuvant therapy followed by pancreatectomy using the National Cancer Database (NCDB 2004-2017). Patients who had <15 nodes examined and those who had ≥15 examined (high ELN) were compared. Univariate and multivariate analyses were performed to determine factors associated with ELN yield. A Cox proportional hazards model was used to identify factors associated with overall survival.

**Results:**

A total of 5,930 patients were included; 58% of patients had ≥15 lymph nodes examined. High ELN was associated with significant improvement in overall survival rates (p<0.004) and perioperative outcomes including post-operative stay (p<0.0001), 30-day unplanned readmission (p<0.028), and 90-day mortality (p<0.001). Patients who were treated at facilities with a high procedure-specific surgery volume were more likely to receive high ELN surgeries than those treated at facilities with a low volume (HR = 2.86[95% CI = 2.36-3.47]).

**Conclusions:**

An ELN yield of ≥15 was a significant measure of surgical quality in this novel population as it was associated with improvements in survival and perioperative outcomes. However, considerable harvest disparities exist at the facility level.

## Introduction

Methods used to assess the quality of surgical interventions in non-metastatic solid tumors should reasonably identify metrics with the greatest curative potential. Current research on lymph node evaluation in pancreatic cancer resection explores the value of resected lymph node yield as a function of survival measures and staging accuracy. While a variety of formats are used to characterize resected lymph nodes, including number of positive lymph nodes (PLN) and lymph node ratio (LNR), much of the reviewed literature agree that higher evaluated lymph node (ELN) yield correlated with increased overall survival (OS) and staging accuracy [1], [2], [3], [4], [5]. Despite evidence also supporting a positive correlation between lymph node yield and survival outcomes in other solid tumor types [6], [7], [8], [9], surgical standards for evaluated lymph node (ELN) minimums are absent. Furthermore, current literature recommendations are highly variable regarding ELN, even among studies of the same cancer type. Tol et al. recommends an ELN yield of at least 15 to ensure accurate staging in pancreatic cancer [1], Vuarnesson et al. suggests 16 [5], and Warschkow et al. advises at least 15-20 for staging and outcome purposes [4]. Based on the findings of these studies, an ELN of 15 or greater was chosen for this paper.

While contemporary literature has been consistent in demonstrating a positive relationship between ELN yield and survival measures, disease staging, and surgical outcomes for patients with pancreatic cancer, the lack of a firm consensus value may also be associated with factors related to treatment. The impact of neoadjuvant therapy on ELN yield has not been previously explored in depth. In a prior study on esophageal cancer, receipt of neoadjuvant radiation alone or chemoradiation was associated with a decreased likelihood of a high ELN compared to no therapy or chemotherapy alone [9]. Treatment at a Community Cancer, Comprehensive Community Cancer, or Integrated Network Cancer programs was also shown to be associated with a lower likelihood of high ELN surgery receipt. Pancreatic cancer patients treated with chemotherapy alone or received treatment at a high-volume facility have also been shown to be more likely to receive high ELN surgeries [3]. However, a more comprehensive examination of these and related variables will be invaluable in the standardization of relevant surgical guidelines.

Current NCCN treatment guidelines suggest patients receive either neoadjuvant or adjuvant therapy alongside surgery for resectable pancreatic cancer [10], and so the establishment of specific minimal ELN yield guidelines for multimodal therapy would be valuable. The aim of this study was to examine the role of high ELN yield (≥15 lymph nodes) during pancreatic cancer resection on long-term oncological survival outcomes in a novel population of pancreatic adenocarcinoma patients who have undergone neoadjuvant chemotherapy alone or chemotherapy and radiation. As a secondary objective, this study sought to identify the variables involved in both the receipt of a high ELN yield and the primary outcome measures of survival.

## Methods

### Study design

This is a retrospective cohort study of patients with non-metastatic pancreatic cancer who received neoadjuvant chemotherapy or chemoradiation and underwent surgery using the National Cancer Database (NCDB, 2004-2017). The NCDB is a joint project between the Commission on Cancer (CoC) of the American College of Surgeons (ACS) and the American Cancer Society. The database is a nationwide, facility-based, comprehensive, clinical surveillance oncology dataset; it captures information from approximately 1500 Commission on Cancer accredited hospitals and more than 70% of all newly diagnosed malignancies in the United States. The data used in the study are derived from a de-identified NCDB file and thus, IRB approval was not required for analysis. The American College of Surgeons and the Commission on Cancer have not verified and are not responsible for the analytic or statistical methodology employed, or the conclusions drawn from these data by the investigator.

Patients were included in the study if a diagnosis of resectable pancreatic adenocarcinoma was confirmed, and if they underwent either neoadjuvant chemotherapy alone or chemotherapy and radiation prior to surgery for their disease. Patients were excluded if their disease was metastatic at clinical staging or if the number of regional lymph nodes examined was unknown.

Facility type designations were based on the designations listed by the National Cancer Database. The definitions of these can be found online [11]. ELN count was based on pathologic lymph nodes using the field “Regional Lymph nodes positive” in the NCDB which “Records the total number of regional lymph nodes that were removed and examined by the pathologist” [12]. High procedure-specific volume in this study was defined as 20 or more pancreatectomies per year, a threshold established according to volume-outcome discussions in the NEJM and the Leapfrog Group [13, 14].

### Statistical analysis

Categorical variables were summarized in terms of frequency and percentages. Comparisons between lymph node yield groups (low yield; <15 vs high yield: ≥15) were performed using Chi-square tests and paired t-tests for statistical significance. Using multivariate logistic regression, potential factors influencing the receipt of a lymph node harvest of 15 or greater were identified. Survival analysis was used to estimate point survival and confidence intervals for each group while log-rank tests were used to assess the null hypothesis that there was no difference in survival probability between the two lymph node yield groups. The proportional hazards model was used to identify the effect of potential factors on survival probability. Statistical analysis was conducted using SAS® Enterprise Guide 8.2: User's Guide (SAS Institute Inc., Cary, NC, USA). Results were considered statistically significant if the p≤0.05. This study was exempt from institutional review board approval and informed consent was waived.

## Results

### Demographics

A total of 5,930 patients who underwent neoadjuvant therapy and surgery were identified (Table 1), while 325,761 patients who did not fit study criteria or who were lacking data on lymph nodes examined or status were excluded from the analysis. The mean age was 64 ± 9.38 years; most patients were 60 to 69 years of age (n=2361; 39.8%) and over 70 years old (n=1706; 28.8%). Male patients were only slightly more common (n=3033; 51.1%) and 87.8% (n=5205) were White. Patients were predominantly treated at academic/research programs (n=3962; 66.8%), followed by treatment at Comprehensive Community (n=1140; 19.2%), Integrated Network (n=683; 11.5%), and Community Cancer (n=145; 2.4%) programs. Patients were overwhelmingly treated at low procedure-specific surgery volume centers (n=5175; 87.3%).

**Table 1:** Demographics.

	Nodes Examined			
	Under 15 (N=2466)	15 or More (N=3464)	Total (N=5930)	P-Value
**Age at Diagnosis**, n (%)				0.0524^1^
40-49	176 (7.1%)	252 (7.3%)	428 (7.2%)	
50-59	572 (23.2%)	863 (24.9%)	1435 (24.2%)	
60-69	962 (39.0%)	1399 (40.4%)	2361 (39.8%)	
≥70	756 (30.7%)	950 (27.4%)	1706 (28.8%)	
				
**Gender**, n (%)				0.4073^1^
Male	1277 (51.8%)	1756 (50.7%)	3033 (51.1%)	
Female	1189 (48.2%)	1708 (49.3%)	2897 (48.9%)	
				
**Race**, n (%)				0.1056^1^
White	2157 (87.5%)	3048 (88.0%)	5205 (87.8%)	
Black	235 (9.5%)	288 (8.3%)	523 (8.8%)	
Other/Unknown	74 (3.0%)	128 (3.7%)	202 (3.4%)	
				
**Insurance Status**, n (%)				0.4556^1^
Not Insured	39 (1.6%)	53 (1.5%)	92 (1.6%)	
Private	1071 (43.4%)	1595 (46.0%)	2666 (45.0%)	
Medicaid	116 (4.7%)	162 (4.7%)	278 (4.7%)	
Medicare	1154 (46.8%)	1543 (44.5%)	2697 (45.5%)	
Other Government	40 (1.6%)	46 (1.3%)	86 (1.5%)	
Unknown Insurance	46 (1.9%)	65 (1.9%)	111 (1.9%)	
				
**Charlson Deyo Score**, n (%)				0.3095^1^
0	1634 (66.3%)	2296 (66.3%)	3930 (66.3%)	
1	639 (25.9%)	928 (26.8%)	1567 (26.4%)	
2	147 (6.0%)	170 (4.9%)	317 (5.3%)	
≥3	46 (1.9%)	70 (2.0%)	116 (2.0%)	
				
**Neoadjuvant Treatment,** n (%)				<.0001^1^
Neoadjuvant Chemotherapy and Radiation	1374 (55.7%)	1407 (40.6%)	2781 (46.9%)	
Neoadjuvant Chemotherapy Only	1092 (44.3%)	2057 (59.4%)	3149 (53.1%)	
				
**Primary Site**, n (%)				<.0001^1^
Body/Tail of Pancreas	410 (16.6%)	419 (12.1%)	829 (14.0%)	
Head of Pancreas	1797 (72.9%)	2724 (78.6%)	4521 (76.2%)	
Other Part of Pancreas	259 (10.5%)	321 (9.3%)	580 (9.8%)	
				
**Procedure**, n (%)				<.0001^1^
Distal Pancreatectomy	333 (13.5%)	306 (8.8%)	639 (10.8%)	
Pancreatectomy, Nos	45 (1.8%)	31 (0.9%)	76 (1.3%)	
Total Pancreatectomy	314 (12.7%)	497 (14.3%)	811 (13.7%)	
Whipple	1774 (71.9%)	2630 (75.9%)	4404 (74.3%)	
				
**TNM Pathologic T Stage**, n (%)				<.0001^1^
T1	375 (15.2%)	385 (11.1%)	760 (12.8%)	
T2	363 (14.7%)	398 (11.5%)	761 (12.8%)	
T3	1603 (65.0%)	2557 (73.8%)	4160 (70.2%)	
T4	116 (4.7%)	115 (3.3%)	231 (3.9%)	
Tis	9 (0.4%)	9 (0.3%)	18 (0.3%)	
				
**Regional Nodes Positive**, n (%)				<.0001^1^
0	1490 (60.4%)	1491 (43.0%)	2981 (50.3%)	
1	428 (17.4%)	580 (16.7%)	1008 (17.0%)	
≥2	548 (22.2%)	1393 (40.2%)	1941 (32.7%)	
				
**Surgical Margins,** n (%)				0.6154^1^
No Residual Tumor	2023 (82.0%)	2824 (81.5%)	4847 (81.7%)	
Residual Tumor	443 (18.0%)	640 (18.5%)	1083 (18.3%)	
				
**Surgical Approach**, n (%)				<.0001^1^
MIS	181 (7.3%)	429 (12.4%)	610 (10.3%)	
Open	1509 (61.2%)	2168 (62.6%)	3677 (62.0%)	
Unknown	776 (31.5%)	867 (25.0%)	1643 (27.7%)	
				
**Facility Type**, n (%)				<.0001^1^
Academic/Research Program	1508 (61.2%)	2454 (70.8%)	3962 (66.8%)	
Community Cancer Program	65 (2.6%)	80 (2.3%)	145 (2.4%)	
Comprehensive Community Cancer Program	530 (21.5%)	610 (17.6%)	1140 (19.2%)	
Integrated Network Program	363 (14.7%)	320 (9.2%)	683 (11.5%)	
				
**Facility Volume,** n (%)				<.0001^1^
Not High Volume	2309 (93.6%)	2866 (82.7%)	5175 (87.3%)	
High Surgery Volume	157 (6.4%)	598 (17.3%)	755 (12.7%)	
				
**Surgical Inpatient Stay,** Days from Surgery				<.0001^2^
Mean (SD)	10.3 (8.53)	9.4 (7.00)	9.8 (7.69)	
Range	0.0, 119.0	0.0, 86.0	0.0, 119.0	
				
**30 Day Unplanned Readmission,** n (%)				0.0275^1^
No Readmission	2280 (92.5%)	3253 (93.9%)	5533 (93.3%)	
Unplanned Readmission	186 (7.5%)	211 (6.1%)	397 (6.7%)	
				
**30 Day Mortality,** n (%)				0.2795^1^
Died	57 (2.3%)	66 (1.9%)	123 (2.1%)	
Not Dead	2409 (97.7%)	3398 (98.1%)	5807 (97.9%)	
				
**90 Day Mortality,** n (%)				0.0006^1^
Died	152 (6.2%)	145 (4.2%)	297 (5.0%)	
Not Dead	2314 (93.8%)	3319 (95.8%)	5633 (95.0%)	

1 chi-Square P-Value.

2 Unequal variance two sample t-test.

Only 58.4% (n=3464) of patient had ≥15 lymph nodes examined (Table 1). Just over half of all patients received neoadjuvant chemotherapy alone (n=3149; 53.1%) and the most common surgical procedure was a Whipple (n=4404; 74.3%). Patients with an ELN <15 had a longer inpatient stay (8.53 vs 7.00 days, p<.0001), a higher rate of unplanned readmission after the procedure (7.5% vs 6.1%, p = 0.0275), and a higher rate of 90-day mortality (6.2% vs 4.2%, p = 0.0006).

### Overall survival (OS)

**Fig. 1:**
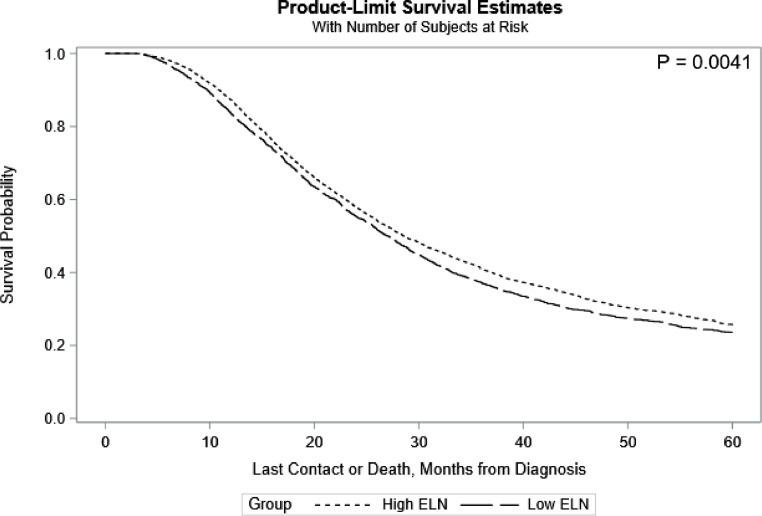
5-Year Survival in both High ELN and Low ELN patients with pancreatic adenocarcinoma who received neoadjuvant therapy.

Overall survival was studied for these populations. The Kaplan-Meier survival curves are shown in Fig. 1. There was a statistically significant difference in overall survival between the two groups. Patients with ≥15 nodes examined had a longer median survival time when compared to those with <15 nodes examined (28.5 [95%CI: 27.6, 29.9] months vs 27.1 [95%CI: 25.9, 28.1] months).

### Factors associated with OS

**Fig. 2:**
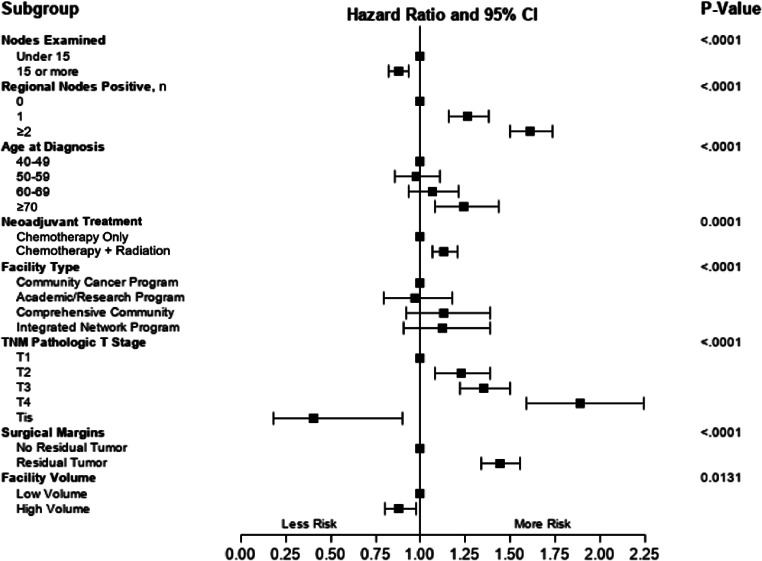
Forest plot of factors associated with overall survival in pancreatic adenocarcinoma patients who received neoadjuvant therapy.

**Fig. 3:**
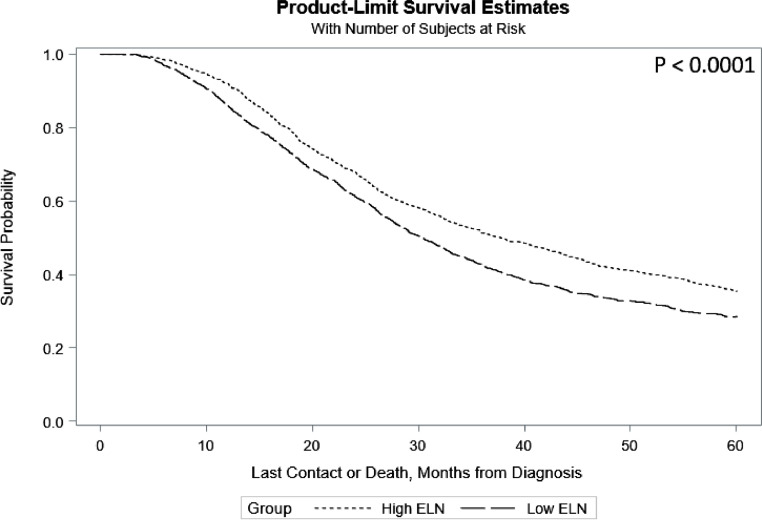
5-Year Survival in both High ELN and Low ELN patients with lymph node negative pancreatic cancer who received neoadjuvant therapy.

A multivariate analysis was performed to investigate potential factors associated with overall survival probability (Fig. 2). Patients with high ELN (≥15 nodes) surgeries saw improved overall survival (HR: 0.879, 95%CI: 0.825, 0.936) compared to those with low ELN (<15 nodes) procedures. Treatment at high volume facilities was similarly associated with increased survival (HR: 0.882, 95%CI: 0.799, 0.974). The presence of positive regional nodes (1[OR: 1.263, 95%CI: 1.159, 1.377], 2+[OR: 1.611, 95%CI: 1.498, 1.732]) was associated with decreased survival, as was the presence of positive margins (OR: 1.442, 95%CI: 1.337, 1.556). Increasing pathologic stage was associated with increasingly poor prognosis (T2[OR: 1.224, 95%CI:1.078, 1.389], T3[OR: 1.349, 95%CI:1.217, 1.495], T4[OR: 1.886, 95%CI: 1.586, 2.244]) were associated with decreased survival. Lymph node yield did affect OS in patients who did not have lymph node metastases. In patients with no positive nodes (n=2981; 50.3%), there was a significant difference in 5-year survival between high and low ELN groups (Fig. 3).

### Factors affecting evaluated lymph node yield

**Fig. 4:**
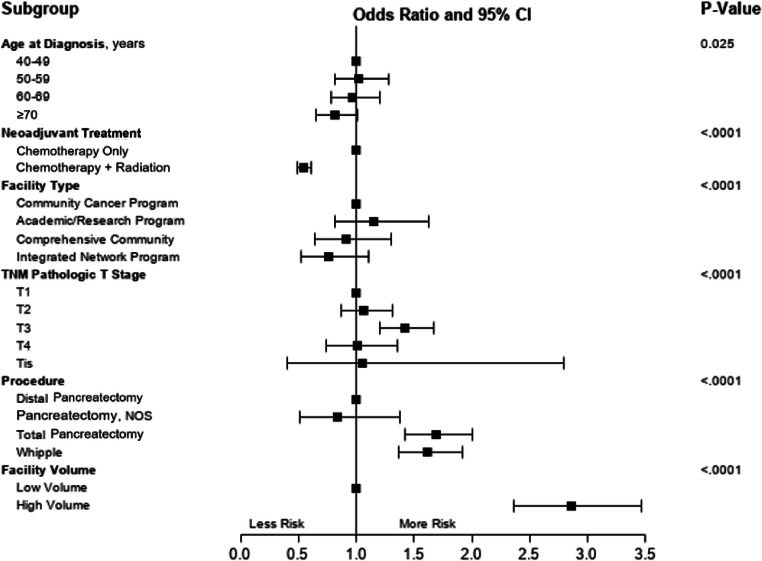
Forest plot of factors associated with ELN in pancreatic adenocarcinoma patients who received neoadjuvant therapy.

A multivariate analysis was performed to evaluate associations with higher ELN (Fig. 4). Patients who underwent radiation in addition to chemotherapy were less likely to have a high ELN (≥15 nodes) compared to those who had neoadjuvant chemotherapy alone (HR: 0.545, 95%CI: 0.489-0.607). Facility type did not influence the ELN yield, however, facility volume did significantly influence the ELN. High volume centers were more likely to have a higher ELN (HR: 2.861, 95%CI: 2.359, 3.469). A pathologic T stage of T3 (OR: 1.418, 95%CI: 1.207, 1.665), receipt of a total pancreatectomy (OR: 1.686, 95%CI: 1.417, 2.006), and receipt of a Whipple procedure (OR: 1.620, 95%CI: 1.366, 1.923) were all associated with high ELN surgeries. The number of regional lymph nodes examined was higher among patients who underwent minimally invasive surgery (MIS) versus patients who underwent open surgery (Table 2).

**Table 2:** Lymph node yield in different surgical approaches.

	Approach			
	MIS (N=610)	Open (N=3677)	Total (N=4287)	P-value
**Regional Lymph Nodes Examined**				<.0001^1^
Mean (SD)	21.5 (12.13)	18.0 (9.94)	18.5 (10.35)	
Median (IQR)	19.0 (13.0, 28.0)	16.0 (11.0, 23.0)	17.0 (11.0, 24.0)	
Range	1.0, 71.0	1.0, 80.0	1.0, 80.0	

1 Wilcoxon rank sum p-value.

## Discussion

This study demonstrates that high ELN (≥15 nodes) is associated with significantly increased overall survival in patients who have undergone neoadjuvant therapy with surgery for pancreatic cancer. In addition to improvement in overall survival, an increased ELN is also associated with improvements in perioperative outcomes. High ELN yield is associated with a decreased length of stay, 30-day unplanned readmission, and 90-day mortality. Even excluding patients with node-positive disease, high ELN still improved OS, indicating the correlation between ELN and patient outcomes reflects benefits of high ELN surgeries beyond just removal of diseased nodes. To our knowledge, this was the first study to investigate the value of high ELN (≥15 nodes) in patients with pancreatic adenocarcinoma who underwent neoadjuvant therapy. The results of this study suggest that an ELN of 15 can serve as a metric for surgical quality amongst this patient population, and all centers should seek high ELN surgeries in pancreatic cancer patients who received neoadjuvant surgery.

The ELN value of 15 was chosen based on the results of Tol et al and Warschkow et al, whose results established the significance of an ELN of 15 in staging accuracy and OS respectively. While our study demonstrates the survival and surgical outcome benefits of an ELN of 15, future work may seek to explore other thresholds for comparable or improved results in this and other patient populations.

This study confirms that nodal harvest rates are a multifactorial variable. ELN was shown to be affected by the type of neoadjuvant therapy used. Pancreatic cancer patients who received neoadjuvant chemotherapy and radiation were less likely to receive surgery with an ELN of 15 or more compared to those who received neoadjuvant chemotherapy alone. A similar relationship has been demonstrated previously in esophageal cancer patients [15]. A study on rectal cancer patients similarly showed that both radiation alone and radiation alongside chemotherapy decreased lymph node yield, due to the shrinkage-effect that neoadjuvant radiation has on nodes [16]. Combination therapy was shown to be associated with decreased overall survival in this study. The continued controversy over the use of radiation in pancreatic cancer patients and absence of discrete standard-of-care recommendations for neoadjuvant therapy indicate the need for further identification of the best practices to be used in the treatment of resectable pancreatic cancer [17], [18], [19], [20].

Surgical approach also contributed to differences in ELN yield in this study. About 70% of patients who underwent MIS and only 59% of patients who underwent open surgery received high ELN surgeries. In addition, the mean number of lymph nodes examined was higher in the MIS group than in the open group. On the surgeon level, it is possible that the minimally invasive approach allows for better visualization for nodal harvest, while the disparity may also be due to differences in preferred approach at the facility level. Prior literature on the effect of MIS versus open on lymph node resection in other solid tumors have demonstrated mixed results. An esophageal cancer study found MIS to improve lymph node yield [21], while two gallbladder cancer studies found MIS was associated with decreased yield or no significant difference at all [22, 23]. While this was not the major focus of this study, the high rate of staging pancreatic disease after it has spread necessitates future study to determine the reason for differences in ELN between surgical approaches.

Treatment at a high-volume facility was also associated with an increased lymph node harvest. Repetition of a procedure likely lends significant benefit to a practitioner's performance. A 2016 study established a high-volume threshold range of 12-31 annual pancreatectomies to reach significant reductions in mortality [24]. The definition of high-volume (≥20 surgeries/year) used in this study fits this criterion as well as those set by the Leapfrog group [14]. High surgery volume on the facility level has been robustly linked to improved short- and long-term outcomes in pancreatic cancer surgery, even when dealing with patient populations characterized by poor prognostic factors [25], [26], [27]. In addition to improvements in overall survival, short-term postoperative mortality, postoperative stay, and higher evaluated lymph node yield were positively correlated with high facility pancreatectomy volume. Although high volume centers have been shown to improve outcomes in cancer patients, not all patients are able to obtain care at high volume centers as is evidenced in this study where 87% of patients were treated at low volume centers. There are several restraints that lead patients to receive care at lower volume centers and not seek out care at high volume centers. Geographic, financial and accessibility restraints as well as overall patient preference play a role in where a patient seeks treatment. All facilities should strive to meet the recommended ELN yield to confer the greatest benefit to the patient.

## Limitations

There are limitations to this study, including the retrospective nature of this database, which precludes any protocolization or standardization among hospitals. There were instances in which critical datapoints in the number of harvested lymph nodes were missing, resulting in the exclusion of those patients from this study. Additionally, the NCDB does not provide data on surgeon-specific metrics, disease-free survival endpoints, and surgical quality measures, all of which would be considered important in a surgical quality study. Lastly, while no database can accurately account for the patient decision-making process in treatment selection, selection of where a patient chooses treatment is relevant in this discussion.

## Conclusion

An ELN yield of ≥15 improves major oncological outcomes in this novel population of patients with pancreatic adenocarcinoma who received neoadjuvant therapy. Factors associated with an increased nodal harvest include pathologic T-stage, procedure type, and treatment at a high-volume center. All centers should seek ELN yields of 15 or greater in order to ensure higher quality surgery.

## Author contributions

All authors made substantial contributions to either the concept and/or design of this study. SW collected and analyzed the data for the present study. KZ, AF, SW, RH, and JB all interpreted the data and drafted and/or edited the manuscript. KY, AF, RH, SW, and JB reviewed the manuscript for errors and provided feedback for changes. All authors approved this version of the manuscript and agreed to be accountable for all aspects of the article.

## Meeting

17^th^ Annual Academic Surgical Congress, February 1-3, 2022 in Orlando, FL

## Sources of Funding

The research did not receive any funding from agencies in the public, commercial, or not-for-profit sectors.

## Declaration of Competing Interests

The authors declare that they have no known competing financial interests or personal relationships that could have appeared to influence the work reported in this paper.
